# Balancing safety, efficacy and cost: Improving rotavirus vaccine adoption in low- and middle-income countries

**Published:** 2011-12

**Authors:** Anant Bhan, Shane K. Green

**Affiliations:** Ethical, Social, Cultural and Commercial (ESC^2^) Program at the McLaughlin-Rotman Centre for Global Health, Toronto, Ontario, Canada

Just over a decade ago, concerns regarding vaccine-related risks led to the demise of the first rotavirus vaccine to enter the market. Licensed in the US in 1998, RotaShield was withdrawn voluntarily in 1999 by its manufacturer, Wyeth, when it was found to be associated with an increased risk of intussusception, a potentially serious and occasionally fatal intestinal obstruction estimated to occur in one case per 10 000 infants given the vaccine (1). This decision was a compelling and controversial one for global health: In seeking to avert rare but serious adverse events caused by the vaccine in the US, it nevertheless vexed efforts to address the staggering burden of diarrheal disease in developing countries. In other words, the potential benefits of a vaccine that might have prevented most of the approximately 500 000 deaths and 1.5 million hospitalizations of infants and young children in Africa and Asia each year caused by rotavirus gastroenteritis (RGE) were overshadowed by risks that some commentators have argued ought to have paled in comparison (2).

Now, in 2011, the rotavirus vaccine landscape has changed, with two licensed vaccines recommended by the WHO Strategic Advisory Group of Experts (SAGE) on Immunization, available for adoption into national immunization programs, and several other vaccines in the development pipeline. And yet, the stark reality is that as of July 2011, only 24 countries (ten of which are lower-middle income, Sudan being the most recent) and no low income countries had adopted rotavirus vaccines into their immunization programs (3), leaving millions of children without access to a crucial tool to prevent rotavirus gastroenteritis (RGE)-associated morbidity and mortality.

The barriers to uptake dotting the landscape have also changed. Safety remains an issue of some concern, particularly in light of emerging data from some post-marketing studies of the currently available rotavirus vaccines that suggest caution. However, considerations of efficacy and cost are assuming more prominence, which is appropriate as each barrier needs to be carefully assessed by decision-makers weighing benefits vs risks. Indeed, the ability to overcome other potential barriers – such as the need to enhance public perception of (and demand for) vaccines, or to stimulate the political will required to commit funding and address implementation challenges – is predicated on rotavirus vaccines demonstrating a favorable balance of benefits to risks. Numbers – efficacy data, calculations of avertable and attributable deaths, vaccine costs – are extremely useful tools, but determinations of a favorable balance (between risk and benefit; between safety, efficacy and affordability) defy simple calculation. Where numbers fail, ethical principles can provide useful guidance. Hence, given the lives that could be saved in the very near future through improved access to rotavirus vaccines, this is an opportune time to re-examine the ethical underpinnings of assessments of benefits vs risks in the context of these vaccines.

## A FINE BALANCE

The prospect of and need for more affordable vaccines that are effective in LMICs provides additional impetus for choosing this moment to reflect on our moral obligations in considering the balance of benefits and risks. Why? Because, while the best case scenario would be for the global public health armamentarium of the near future to comprise a suite of efficacious, safe and affordable rotavirus vaccines that can be rolled out as appropriate and feasible across all jurisdictions, the distinct possibility exists that new rotavirus vaccines will not hit the trifecta of being more affordable, equally or more efficacious in all settings, and equally safe or safer relative to those available today. What place, if any, is there for vaccines with equal or superior efficacy in LMICs that are more affordable, but even marginally less safe, than those currently available? Recent assertions of favourable balance of benefits to risk in rotavirus vaccination programs, while a welcome change from the dialogue ten years ago (2), nonetheless focus on the safety and efficacy of the vaccine rather than its *effectiveness* in a real world setting, which is where cost and affordability come into the picture.

All else being equal, a lower cost - and therefore more readily accessible vaccine - would demonstrate greater effectiveness than a higher priced vaccine. But how significant does the difference in effectiveness need to be to justify the use of the more affordable vaccine, if it carries a slightly elevated risk of intussusception relative to its more expensive – and thus arguably less effective – counterpart? In answer, we can appeal to principles of public health ethics and suggest that it may well be possible that such a marginally less perfect vaccine could, by virtue of being more affordable and thus more accessible, *promote greater good* through enhanced *effectiveness* in the face of clear *necessity* within a given context, and its use therefore ethically defensible.

## SAFETY

For regulators, policy makers, global health advocates and families, the safety of rotavirus vaccines has long been a paramount concern. This is understandable, given our shared societal and moral obligation to avert preventable harm, which includes minimizing and/or mitigating harms from vaccination. The increased risk of intussusception caused by Rotashield was deemed excessively high and its withdrawal was a prudent move in the US, given its relatively low burden of diarrheal disease caused by rotavirus – some 20-60 RGE-linked deaths annually (4). However, failure to further test and deploy the vaccine in developing countries over the past decade may have cost millions of lives in those countries, in which the staggering disease burden is about 10 000 times greater than in the US (2). One need not overlook - or even downplay - the significance of the deaths that might have been caused by widespread Rotashield vaccination in high disease burden countries. However, when weighed against the potential for the vaccine to save hundreds of thousands of lives each year globally, the moral obligation to avert preventable harm should rightly have tipped the balance decidedly in favor of vaccination.

The issue of intussusception persists, but is now set against increasing evidence of benefit in LMIC settings with high disease burden. In pre-licensure studies involving more than 60 000 infants each, the currently available vaccines, RotaTeq (Merck) and RotaRix (GlaxoSmithKline [GSK]), were shown to offer protection from rotavirus infection to children for the first two years of life without evidence of increased risk of intussusception among the study populations, meriting FDA licensure in 2006 and 2008, respectively (5). But post-marketing studies are still ongoing, and thus the complete data necessary to conduct a comprehensive assessment of safety among larger sample sizes across diverse populations are not yet available. In the past year, however, important data from LMICs have begun to emerge: one recent study in Jamaica found rotavirus vaccine to reduce health care utilization attributable to RGE without increased risk of intussusception (6). Other post-marketing studies from Australia, Brazil and Mexico showed persistent link between rotavirus vaccines and increased risk of intussusceptions (7,8). Specifically, the studies in Mexico and Brazil found vaccine-attributable intussusceptions in one in 51 000, and one in 68 000 infants, respectively, vaccinated with the monovalent rotavirus vaccine (i.e., RotaRix); at the same time, the vaccine prevented 80 000 hospitalizations and 1300 deaths otherwise caused by RGE. On the strength of these numbers, both the study authors and the editorialist in the *New England Journal of Medicine* were unequivocal in their assessments that rotavirus vaccination has a favorable ratio of benefit to risk; in fact, the editorial also opined that a “favourable ratio would probably also have been present with [RotaShield]” (9), demonstrating the extent to which the dialogue around benefits and risks has shifted to account for the fact that even imperfectly safe vaccines can nonetheless be responsibly used to save many lives where the need is the greatest.

It is yet unclear whether these data adequately represent the risks to populations across all LMICs, and whether health systems strengthening will still be needed before adequate post-vaccine surveillance programs can be meaningfully implemented in many LMICs where public health monitoring is often insufficient and/or ineffective (10). Nonetheless, the WHO recommends that the absence of such post-marketing surveillance at the onset should not be an obstacle to introducing rotavirus vaccines (11). In the meantime, the concept of *progressive realization*, which advocates a step-wise approach to achieving socially important goals, can be usefully applied here to help guide national and regional policy making to gradually enhance health systems’ internal capacity for post-marketing surveillance. There is a lot to learn in this regard from initiatives, such as the Safety of New Vaccines (SANEVA) network developed in 2006 among five countries in Latin America (Argentina, the Bolivarian Republic of Venezuela, Brazil, Mexico and Panama), where one of the first foci has been to monitor cases of intussusceptions following the introduction of rotavirus vaccines in member countries (12).

What remains unclear, however, is just how imperfectly safe a vaccine could be within a given disease burden context to still have a favorable balance of benefit to risk? Here, numbers fail to provide adequate guidance: even if we were to accept that, in a country with a high disease burden, a ratio of one vaccine-linked intussusception in 51 000 vaccinated infants is favorable, and 1 in 10 000 ‘probably favorable,’ it is not obvious how to choose the appropriate bar below which it becomes probably or outright *un*favorable. Principles of public health ethics – notably including *effectiveness, necessity, and promoting the greater good* (13,14) – can provide useful guidance.

**Figure:**
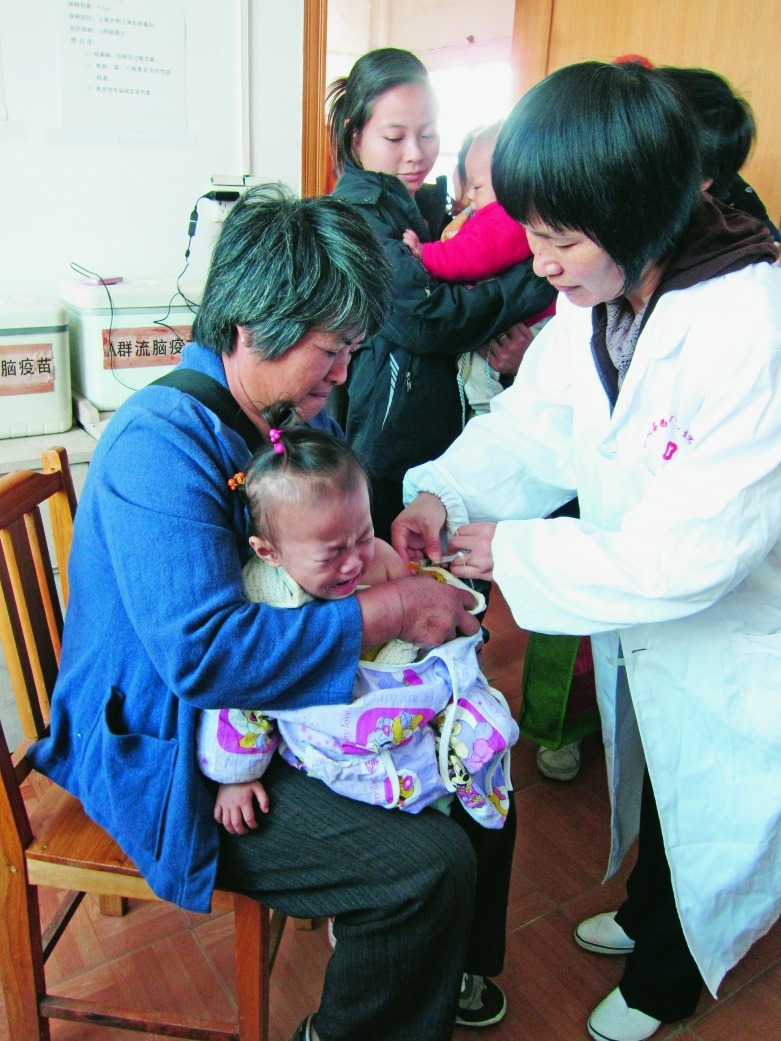
Photo: Courtesy of Dr Kit Yee Chan, personal collection

The principle of *effectiveness* requires that if other moral considerations (e.g., *do no harm*) are to be infringed, evidence of real-world effectiveness in improving public health must be present within a certain context. At the same time, the principle of *necessity* allows for conflict across moral principles, but holds that no other method of achieving a particular ‘end’ would have less conflict with other moral considerations (13,14). Because the goal of public health is to maximize the welfare of a population – *promoting the greater good* – some element of risk in a public health program (e.g., risk of intussusception) can be deemed morally acceptable if the program and its anticipated health and societal benefits are seen by decision-makers within a population – politicians, public health officials, and families alike – as satisfying the principles of *effectiveness* and *necessity*. Oral Polio Virus (OPV) mass administration, for example, has been linked to an increase in cases of acute flaccid paralysis due to vaccine-derived polio virus; nonetheless, because of the health and social value of quelling the spread of polio in those few regions in which it remains endemic, OPV has remained the main vaccine of choice in mass campaigns to control polio in countries like India (15). The emphasis on *effectiveness* and *necessity* as determined within communities and/or populations highlights the importance of giving due consideration to local context, including disease burden; for example, from a public health ethics perspective, with relative disease burden seemingly overlooked in the decision to withdraw Rotashield, the assessment of risks vs benefits – based solely on numbers in one context, but not in others – was flawed, and terribly costly.

## EFFICACY

Experts continue to be flummoxed by data showing that rotavirus vaccines demonstrate lesser protective rates of efficacy in LMICs as compared to 85-98% seen in high-income countries (16). This phenomenon, often termed the ‘tropical barrier’, is not yet fully understood. Researchers have implicated factors such as mucosal immune dysfunction brought about by repeated infections, poor nutrition (17), and higher titres of IgA and neutralizing activity in breast milk (18). Recent data have seemingly assuaged previously articulated concerns about the potential for concomitant administration of multiple oral vaccines (such as OPV and rotavirus vaccine) to contribute to the reduced efficacy (19). Efficacy may be further compromised in some LMIC settings, like India, in which the available rotavirus vaccines do not provide protection against all the prevailing strains (20). Reduced efficacy alone, however, should not deter policy makers in LMICs from accelerating their adoption, as the potential public health benefits – lives saved and infections averted – are still highly significant. A recent analysis assuming vaccine efficacy of 50% in a national rotavirus immunization program implemented in India estimated that it would still prevent approximately 44 000 deaths, 293 000 hospitalizations, and 328 000 outpatient visits annually, which would avert US $20.6 million (€ 15.7 million) in medical treatment costs for the country (21).

While there is a need for continued fundamental and applied research to better understand and improve the efficacy of rotavirus vaccines in LMICs (19), it is critical to also acknowledge that the real-world *effectiveness* of the vaccines in LMICs would depend not only on efficacy, but also on a number of other factors, including access to health care.

## COST

At the moment, there appears to be some consensus that the potential for rotavirus vaccines to save hundreds of thousands of children’s lives outweighs their still uncertain, but potentially modest increased risk of intussusception, and variable efficacy in LMICs. However, in order for the benefits to be realized, these – or other – rotavirus vaccines must be affordable enough to reach those whose lives they are expected to save. It is therefore unsurprising that safety and efficacy concerns appear now to be matched by concerns about the affordability of the vaccines for LMICs (22-24).

In June 2011, GSK and Merck took a laudable step towards addressing this barrier by announcing that they would make their vaccines available to the GAVI Alliance at significantly reduced prices for use in the 72 LMICs currently eligible to receive GAVI support for rotavirus vaccines (25,26). However, even at the drastically reduced rates, the cost of vaccinating entire populations of children in many LMICs may remain very challenging, or even prohibitive. By exceeding its funding targets at its recent pledging conference (27), GAVI has proven capable of galvanizing funders’ support for vaccines. Still, a successful global roll-out of rotavirus vaccines will require not only the pledged support, but also much more, including LMIC governments’ commitments to co-financing. Moreover, other authors have recently noted that the uncertainty around poor countries’ capacity to sustain their access to affordable vaccines in the post-GAVI period will probably remain the largest for rotavirus vaccines (28).

Bridging the funding gap will likely depend on the introduction by innovative developing world vaccine manufacturers of new, markedly less expensive rotavirus vaccines, several of which are under development. Farthest along are candidate vaccines from Bharat biotech (Phase III) (29) and the Serum Institute of India (Phase II) (30). In a remarkable display of optimism, Bharat biotech has already committed to making its vaccine available for US$ 1 (€ 0.8) per dose, expecting licensure in India in 2014 and WHO prequalification the following year (31). LMIC development and manufacturing of low cost alternatives could dramatically alter the landscape – much as it did for HIV treatment through the manufacture of low cost generic antiretroviral drugs over the past decade – not least by spurring developed world manufacturers to further reduce the prices of their products. As prices fall, the accessibility of rotavirus vaccines and their potential to prevent RGE-related mortality and morbidity in LMICs will rise.

## MOVING FORWARD

The rotavirus vaccine landscape is much different today than it was a decade ago. It will continue to evolve for the foreseeable future with the emergence of new data and vaccines. Efforts to mitigate risks will continue through improved post-marketing surveillance, better health systems and safer vaccines. Newer vaccines will improve on efficacy in low-resource settings by incorporating knowledge about factors predisposing enteric vaccines to the tropical barrier. Global funding agreements, advocacy and the marketplace entry of vaccines developed by innovative southern companies will bring down the cost of vaccines.

While we cannot be certain of how the safety, efficacy and cost profiles of rotavirus vaccines will change over time, it is still likely that no single vaccine will demonstrate the perfect combination of total safety, complete efficacy and sufficient affordability for use in all contexts where it is needed. Regulators and public health officials in LMICs will need to continue to assess the balance of benefits vs risks in making decisions to approve and/or adopt rotavirus vaccines in their respective jurisdictions. Because *effectiveness* is affected not only by how safe and efficacious a vaccine is, but also whether it is accessible, we contend that such assessments, normally based on safety and efficacy alone, must also include affordability. Furthermore, we suggest that this rationale should not only apply to rotavirus vaccines, but also to other vaccines targeting diseases that disproportionately impact populations in LMICs, such as pneumococcal vaccines. Neglecting to do so in the case of rotavirus vaccines would – once again – keep an effective and life-saving public health intervention from those who need it the most, and constitute a moral failure in global health.
